# Gait Transitions in Human Infants: Coping with Extremes of Treadmill Speed

**DOI:** 10.1371/journal.pone.0148124

**Published:** 2016-02-01

**Authors:** Erin V. Vasudevan, Susan K. Patrick, Jaynie F. Yang

**Affiliations:** 1 Rehabilitation Research and Movement Performance (RRAMP) Lab, Health and Rehabilitation Sciences, School of Health Technology and Management, Stony Brook University, Stony Brook, New York, United States of America; 2 Department of Physical Therapy, University of Alberta, Edmonton, Alberta, Canada; 3 Neuroscience and Mental Health Institute, University of Alberta, Edmonton, Alberta, Canada; Scientific Institute Foundation Santa Lucia, ITALY

## Abstract

Spinal pattern generators in quadrupedal animals can coordinate different forms of locomotion, like trotting or galloping, by altering coordination between the limbs (interlimb coordination). In the human system, infants have been used to study the subcortical control of gait, since the cerebral cortex and corticospinal tract are immature early in life. Like other animals, human infants can modify interlimb coordination to jump or step. Do human infants possess functional neuronal circuitry necessary to modify coordination *within* a limb (intralimb coordination) in order to generate distinct forms of alternating bipedal gait, such as walking and running? We monitored twenty-eight infants (7–12 months) stepping on a treadmill at speeds ranging between 0.06–2.36 m/s, and seventeen adults (22–47 years) walking or running at speeds spanning the walk-to-run transition. Six of the adults were tested with body weight support to mimic the conditions of infant stepping. We found that infants could accommodate a wide range of speeds by altering stride length and frequency, similar to adults. Moreover, as the treadmill speed increased, we observed periods of flight during which neither foot was in ground contact in infants and in adults. However, while adults modified other aspects of intralimb coordination and the mechanics of progression to transition to a running gait, infants did not make comparable changes. The lack of evidence for distinct walking and running patterns in infants suggests that the expression of different functional, alternating gait patterns in humans may require neuromuscular maturation and a period of learning post-independent walking.

## Introduction

The spinal locomotor circuitry of quadrupedal animals is capable of coordinating a remarkably wide repertoire of gait patterns in response to changes in afferent input [1–4]. For instance, spinalized cats supported over a moving treadmill can step at a range of speeds and can even transition to different gaits, such as trotting or galloping, as the speed increases [5]. These animals can also coordinate their hind limbs to walk at different velocities on a split-belt treadmill. A common rhythm was maintained between the legs up to a two- to three-fold speed differential; at more extreme differentials, the limb on the fast belt would take two, three, or four strides during one stride cycle of the slow limb [5]. In addition, spinalized cats and rats are capable of modifying their gait patterns to avoid or accommodate a perturbation [6–9]. It is, therefore, clear that spinal networks in quadrupeds can recognize changes in the environment via afferent input and coordinate locomotor behavior appropriately to maintain forward progression.

Evidence from people with spinal cord injury suggests that spinal networks for bipedal human locomotion are also responsive to changes in afferent input [10], although whether this flexibility exists to the same degree as in quadrupeds is unclear. One informative model used to study the subcortical control of bipedal walking is the human infant. When supported over a moving treadmill or over ground, infants show a stepping behavior at birth that is thought to be largely independent of volitional control [11–14], since anencephalic infants also show stepping [15]. In typically developing infants, the neocortex and descending motor tracts are immature. The motor cortex is largely unmyelinated at birth and rapidly grows and develops over the first two years of life [16]. ^18^FDG positron emission tomography shows that glucose utilization increases in the sensorimotor cortex until approximately one year of age, when a pattern resembling that of an adult is seen [17]. Axons comprising the corticospinal tract are estimated to be 10 times smaller in diameter, and thus 10 times slower, than axons in the adult [18], and histological evidence suggests that adult-like myelin does not appear until 2 years of age [19–21]. Development of myelin occurs in a rostral-to-caudal direction, thus myelination of the tracts extending to the lumbar spine occurs later in development than myelination of cervical spine pathways. Positive Babinski reflexes, indicative of limited transmission in corticospinal tracts, persist until ~18 months of age [22]. As in other animals, sensory input shapes the motor pattern for stepping in infants, enabling them to step at different treadmill speeds [12], in different directions [23], on a split-belt treadmill [24, 25], and during perturbations imposed by external obstacles [26, 27]. The extent to which infants can also coordinate different forms of bipedal gait, like walking and running, in response to changes in afferent input is, however, unclear.

Interestingly, gait transitions with increasing locomotor speed in quadrupeds are mostly accomplished by altering coordination between the limbs (interlimb coordination), with minor changes in the coordination within a limb (intralimb coordination} [5, 28, 29]. Several studies have shown that human infants are similarly capable of generating different forms of interlimb coordination [24, 30–35]; individual infants show preferences for alternating (i.e., stepping) or synchronous (i.e., jumping) interlimb coordination which can be influenced by training [31]. Speed-related gait transitions in human adults (i.e., walking to running) are not associated with changes in interlimb coordination, but rather with changes in intralimb coordination. For example, there is a sudden increase in the angle of maximum knee flexion during swing at the walk-to-run transition [36, 37] and a large (~25%-40%) reduction in the support length (i.e., the distance travelled during the stance phase) [36]. Additionally, there are significant shifts in the displacement of the center of mass during running, as compared to walking, which reflect a switch from an ‘inverted pendulum’ gait (walking) to a spring-like mode of progression (running–for review, see [38, 39]). These changes in adult gait from walking to running are quite different from the typical quadrupedal gait transitions that are characterized by changes in interlimb coordination.

In this study, we evaluated whether infants possess functional neuronal circuitry necessary to modify coordination *within* a limb (intralimb coordination) in order to generate distinct forms of alternating bipedal gait, specifically walking and running. A previous study by our group documented that several infants were able to step at treadmill speeds exceeding 1.0 m/s (comfortable walking speed for adults), and one infant continued stepping up to 2.0 m/s (near the adult walk-to-run transition) [24], demonstrating that at least some infants could exceed the speed at which a transition to running should occur. To a casual observer, these infants also looked like they were running (S1 Video). The current study takes a more systematic approach to determine how infants modify intralimb coordination to accommodate such a wide range of speeds, and whether infants are truly capable of generating distinct walking and running pattern at different speeds.

## Materials and Methods

### Subjects

A total of 28 infants who performed successful stepping at two or more different speeds during the experimental trials were included in analysis. The infants ranged in age from 5.8–11.8 months (average age = 9.7±1.3 months) and were unable to walk independently. Infants were recruited through local health clinics. Prior to the experiment, parents were instructed to practice the stepping response with their infant at home, as described in Yang et al. [12]. Practice consisted of the parent supporting the infant to stand on a flat surface, like a table or floor. The parent was instructed to move the infant forward until the infant took at least one stride. Once the infant was able to perform ten consecutive strides (as assessed by a parent), an experiment was scheduled. Seventeen adults (age 22–47 years; mean ± s.d. = 29.1 ± 7.6 years) were recruited for comparison to mature running. Ethical approval for this study was obtained through the Health Research Ethics Board, University of Alberta and Alberta Health Services, Edmonton and through the Institutional Review Board at Stony Brook University. Informed and written consent was obtained from adult participants or from a parent or legal guardian prior to the experiment. All experiments were conducted in accordance with the Declaration of Helsinki for experiments on humans.

### Instrumentation

Most infants (n = 25) were studied while walking on a custom-built split-belt treadmill. The two treadmill belts were driven by separate motors, which can be electrically coupled to run at the same speed (tied-belt) or uncoupled to run separately (split-belt). In this experiment, the two belts were always at the same speed (tied-belts). Vertical ground reaction forces were measured with force plates located under each treadmill belt. A Plexiglas partition (15 cm in height) was placed between the two belts to ensure that the infant’s legs remained on separate belts. Three infants were studied while walking on an adult treadmill without split belts (Gaitway treadmill system, Kistler Instruments, Amherst, NY, USA); ground reaction forces were not reported for these three infants. Adult walking and running were studied on a split-belt treadmill (Woodway USA Inc., Waukesha, WI) run with both belts moving at the same speed (i.e. tied-belts). A partition between belts was not used for adult studies since the adult split-belt treadmill was not instrumented with force plates.

**Fig 1:**
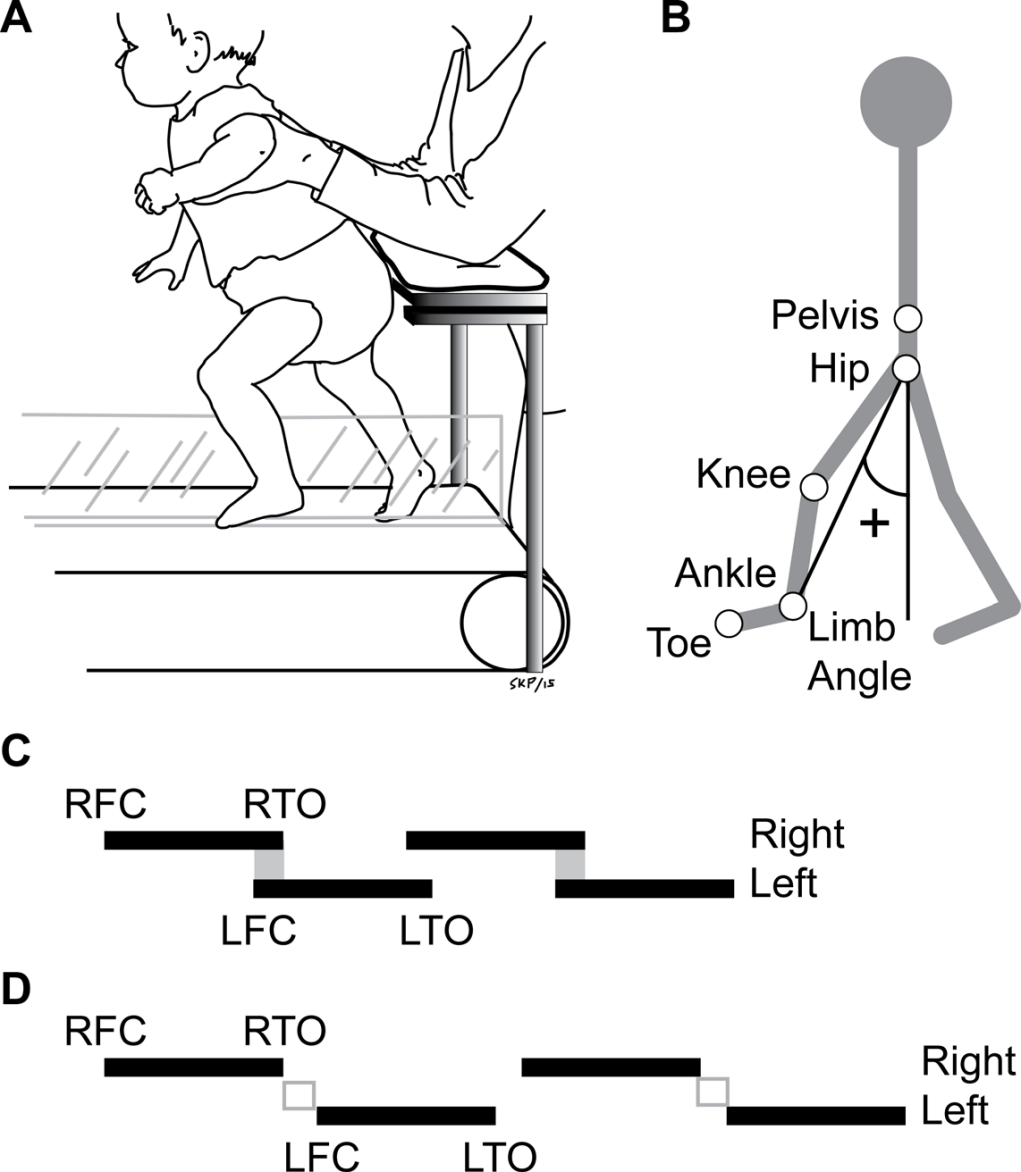
Experimental setup and explanation of measures. **(A)** Infants were held by an experimenter or parent in the center of the treadmill, with one leg on either side of a Plexiglas partition separating the belts. Forearm supports were provided for the person holding the infant to limit the possibility of imposing movements on the infant. **(B)** Stick figure showing placement of markers in infants and adults. Limb angle is defined as the angle formed between a vector from the hip to ankle and a vertical line. Positive limb angles denote limb flexion. **(C)** Example stride cycles with periods of double-support. Solid lines show stance phase, from foot contact (R or LFC) to toe-off (R or LTO). Spaces between lines indicate swing phase. Periods of double-support at the end of right stance are shown by grey boxes. **(D)** Example stride cycles with periods of flight (shown by open grey boxes).

Infant kinematic data were either provided by electrogoniometers (Biometrics Ltd., Newport, UK) placed over the right knee and hip joints (n = 16), or by digitized video (n = 12 –Vicon Motus, Denver, CO). To perform the video analysis, a digital video camera (Elura 50, Canon, Tokyo, Japan) recorded the movement of the right side (sagittal plane) at 30 frames per second. Markers were placed over the midline of the trunk above the iliac crest (pelvis), greater trochanter (hip), knee joint line (knee), lateral malleolus (ankle), and fifth metatarsal (toe) on the right leg (Fig 1B). The infants were dressed in black tights to enhance the contrast of the white markers (2 cm in diameter). Adult motion capture was achieved using an Optotrak system (Northern Digital, Waterloo, ON, Canada), which sampled at 100Hz. Infra-red emitting markers were placed bilaterally at the same landmarks as shown in Fig 1B.

Video, Optotrak, and analog signals were synchronized by a custom-made digital counter that generated a 5V pulse (1Hz) and advanced an LED display (resolution 10ms) in view of the camera. In addition, an output pulse from the Optotrak indicated the timing of the first and last frame from that system. Force-plate signals were low-pass filtered at 30Hz and analog-to-digital converted at 250Hz (Axoscope, Molecular Devices, Sunnyvale, CA).

### Experimental procedures

The experimental session for each infant and adult participant was approximately 30 min—1 hour. Infants played on a mat while the transducers were applied. Once the infant was fully instrumented, (s)he was placed on the treadmill with the feet on the moving belt. Infants who walked on the split-belt treadmill were placed over the center of the two force plates, with one leg on either side of the partition of the split-belt treadmill, with force plates measuring ground reaction forces of each leg (Fig 1A). The infant was held under the arms from behind by one of the experimenters or a parent. Forearm supports were provided for the person holding the infant to limit the possibility of imposing movements on the infant. The infant was encouraged to support as much body weight as possible throughout the stride cycle. The experimenter/parent holding the infant was instructed to only lift the infant up in order to prevent a fall or at the end of a trial. Data analysis was terminated at the point when lifting occurred.

For infant participants, each experiment began with a trial at a slow-to-moderate speed (0.3–0.6 m/s) to allow the infant to become accustomed to the treadmill. The order of subsequent speeds was chosen to test the widest range of speeds feasible for each infant. We attempted to get at least one successful trial at < 0.3 m/s and, with infants who were happy to perform several trials, we increased the treadmill speed until stepping stopped. Usually belt speed was changed in between trials, when the infant was not in contact with the treadmill. Occasionally, belt speed changed mid-trial; in these instances, any stepping that occurred during the acceleration or deceleration phase were not included in analysis. Experiments ended when sufficient data were collected, when the infant became irritable, or when (s)he stopped stepping (i.e. let the feet drag behind and/or no longer supported any body weight).

In separate trials, seven adult participants were asked to either walk or run at a treadmill speed of 2.0 m/s, which is near the walk-run transition speed. An additional four adults were asked to walk and run across a range of overlapping speeds (walk speeds: 0.5, 1.0, 1.5, 2.0 m/s; run speeds: 1.0, 1.5, 2.0, 2.5, 3.0 m/s). In a final set of adult experiments, the effects of body weight support were evaluated in six adults who walked or ran across the same speed range listed above (0.5–3.0 m/s). An external body weight support and harness system (ZeroG dynamic gait and balance training system, Ashburn, VA, USA) was used to unload each participant by 10% and 50% of his/her body weight (i.e., 10% or 50% body weight support or BWS).

### Data analysis

For infant participants, the video record was reviewed off-line to identify successful trials. A trial was considered successful if it included at least one sequence of six consecutive, continuous steps (i.e. three strides on each side). Continuous stepping was defined as rhythmic movement of the legs, without noticeable pauses in movement based on visual inspection (e.g., due to dragging toes at end stance or due prolonged flexion of the limbs at end swing). A “stride” was defined as a cyclic movement that included placement of the foot ahead of the hip marker. In other words, all strides included in analysis showed active flexion of the limb. Interlimb coordination during stepping sequences did not have to be strictly alternating (S1 Fig); however, sequences of bouncing (synchronous interlimb coordination) were excluded in order to focus our analysis on changes in intralimb coordination during stepping at different speeds. Relative proportions of synchronous and alternating interlimb coordination during infant treadmill locomotion have been reported elsewhere [31].

Once the stepping sequences were identified, the onsets of stance and swing phases were determined from the video. The onset of stance was defined as the time that the foot made contact with the treadmill and started moving backwards with the treadmill belt. The onset of swing was defined as the time that the toe marker changed from moving backward (stance phase) to moving forward (swing phase). Digitized force plate and electrogoniometer data were also used to assist in the identification of stance and swing phases. Sometimes discrepancies between the video and analog data occurred due to toe drag at the beginning of swing, which resulted in a non-zero force plate recording. In such cases, video of the experiment was used to identify stance and swing phases.

For most of the adult participants (n = 10), the onset of stance and swing was identified using force sensitive resistors (FSRs) placed under the heel and first metatarsal; signals from the FSRs corresponded to when the foot was in contact with the ground. FSRs were not used for seven adult participants who were tested while walking and running under normal loading conditions; instead, the onset of stance and swing was determined by maximum and minimum limb angle values, respectively. Limb angle is defined as the angle formed between a vector from the hip to ankle marker relative to the vertical (Fig 1B). Limb angle is positive when the limb is flexed forward of the hip (e.g. foot contact), 0° indicates a neutral position under the trunk, and negative indicates limb extension. Comparison of limb and knee angle traces from adult data in which limb angle was used to define ground contact and limb and knee angle traces from adult data in which FSRs were used to define ground contact indicated that both methods provided a comparable estimate of the onset of stance and swing.

For both infants and adults, once the onset of swing and stance were identified in the digitized data, data from each of these strides were analyzed using custom-written programs in MatLab (MathWorks, Natick, MA). The phase relationship between the two legs during stepping was quantified as the time of right side foot contact, relative to the left side stride cycle, and expressed as a percentage. Therefore, values near 50% would indicate alternating stepping and values near 0 or 100% would indicate an in-phase pattern, such as jumping. The duration of double support (or flight) was calculated as the time interval between end-stance on the right side and onset of left foot contact (depicted by grey boxes in Fig 1C and 1D). A period of flight was indicated by a negative value, indicating that foot-contact occurred after end-stance on the contralateral leg (Fig 1D). To estimate loading during infant trials, the vertical ground reaction force was averaged across the stance phase and normalized to the infant’s body weight.

Maximum knee flexion during swing phase and the support were calculated for each stride and averaged across strides within each trial at a constant speed. Support length was calculated as the distance traveled by the ankle marker during stance phase, as the foot was moved backwards by the treadmill belt [36]. For this analysis, infant trials were defined as “walking” or “running” based on whether a majority of strides showed a period of flight (i.e., trials in which >50% of strides showed a period of flight were classified as “running”).

Since many infant trials included some strides with double support phases and some strides with flight phases, an additional analysis was performed to more specifically examine differences between strides with a period of double support and flight. Joint angle excursion and center of mass displacement over the stride cycle were examined in a subset of 10 infants from whom we collected motion tracking data (from digitized video), and who also performed at least one successful trial at a “transition speed”, defined as a speed at which 30–70% of strides showed a period of flight. Data from double-support and flight strides taken at the same speed (i.e. within the same trial) were averaged separately. These data were compared to averaged stride data from adults who walked and ran at the same speed. To compare timing shifts in the center of mass displacement between this subset of infants and adults, the height of the right hip marker was used as an estimate of center of mass. Within subjects, the vertical position of the hip marker was normalized to average height of the marker across each stride. This was done so that displacement from the mean position could be compared across subjects of different heights. A cross-correlation analysis was performed within-subjects on these hip height signals from averaged double-support and flight strides. Timing changes were quantified as the phase lag at peak cross-correlation. Values near zero indicate little shift in timing. These analyses were also performed to compare adult data between differing body weight support conditions.

### Statistics

Pearson correlation coefficients were used to evaluate the relationship between gait speed and kinematic parameters (max knee flexion and support length), and between gait speed and body weight support in infants. To evaluate differences between kinematic parameters of strides taken with a period of double-support (i.e., walking trial in adults) versus flight (i.e., running trial in adults), t-tests were used. Two-way repeated measures ANOVAs (two body weight conditions x two forms of gait) compared kinematic parameters during adult walking and running trials performed under conditions of 10% and 50% body weight support. Significance was determined as ρ<0.05.

## Results

Infants were capable of stepping at speeds ranging between 0.06–2.36m/s. Alternating stepping patterns were preserved across speeds; on average, the phase relationship between the two legs during infant stepping was 51.45 ± 5.35%, indicating that the right side initiated stance approximately half-way through the left side stride cycle. We also found that infants supported, on average, roughly half of their own body weight during stepping (47.0 ± 16.9%). There was no significant relationship between percent body weight support and treadmill speed (r^2^ = -0.04).

**Fig 2:**
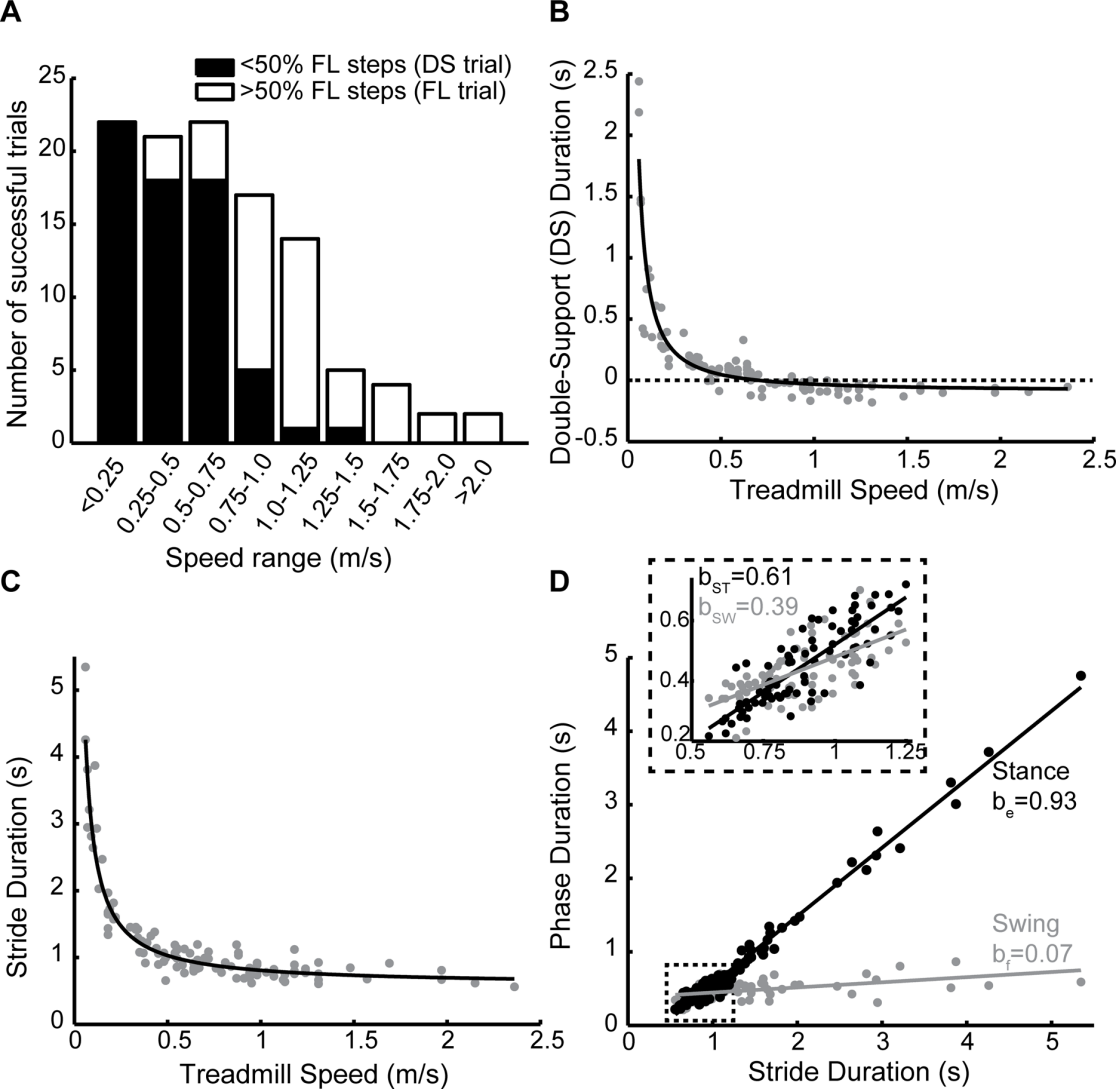
Adaptation to treadmill speed: stride cycle modifications. **(A)** The number of trials that were analyzed at each treadmill speed is shown by the height of each bar. Trials in which the majority of strides had a period of double support are shown in black; trials with a majority of flight strides are in white. **(B)** Average double support duration for each successful trial shown in (A). Negative values indicate flight. Data were fit with a second order power function (*ax*^*b*^ + *c*). **(C)** Average stride duration for each successful trial; data were fit with a second order power function, as described previously [11]. **(D)** Average durations of stance (black) and swing (grey) change linearly with stride duration–the slope (b) of each line is shown beside the plots. Inserted plot shows data from the highlighted region (stride duration = 0.55–1.25s; lines were re-fit to these data and recalculated slopes are shown in insert).

Fig 2A shows the number of successful trials across different speeds. For reference, adult preferred walking speed is approximately 1.1–1.2m/s [40, 41] and the walk-run transition occurs around 1.88–2.35m/s [42–45]. In Fig 2A, black bars indicate the number of trials where the majority of strides showed a period of double-support (see also Fig 1C); white bars indicate trials where the majority of strides had a period of flight (see also Fig 1D). Overall, most infants stepped at speeds of up to 1.25m/s. Not as many successful trials were observed at speeds exceeding 1.25m/s. This is likely due to a combination of factors, such as fatigue, irritability, and an inability to continue stepping at increasing speeds. We were unable to define a maximum limit of stepping for individual infants due to this complex interaction between variables affecting the infants’ willingness to continue participation. Nonetheless, many infants showed periods of flight, a characteristic of running in adults, at speeds in excess of 0.75–1.0m/s (Fig 2B). Since many successful trials were obtained in which infants walked with a “walking” gait (double-support strides) and a “running” gait (flight strides), we were able to compare and contrast these two forms of progression.

Overall, infants adapted to faster treadmill speeds by incorporating a period of flight into the stride cycle (Fig 2B) and decreasing stride duration (Fig 2C). Fig 2D shows whether changes in stride duration are explained primarily by changes in the duration of stance (extension) or swing (flexion) phase–note that longer stride durations are associated with slower walking speeds. At slower speeds, changes in stride duration were dominated by changes in the duration of stance phase (Fig 2D). However, at faster speeds, swing phase increased its dominance (see insert in Fig 2D).

**Fig 3:**
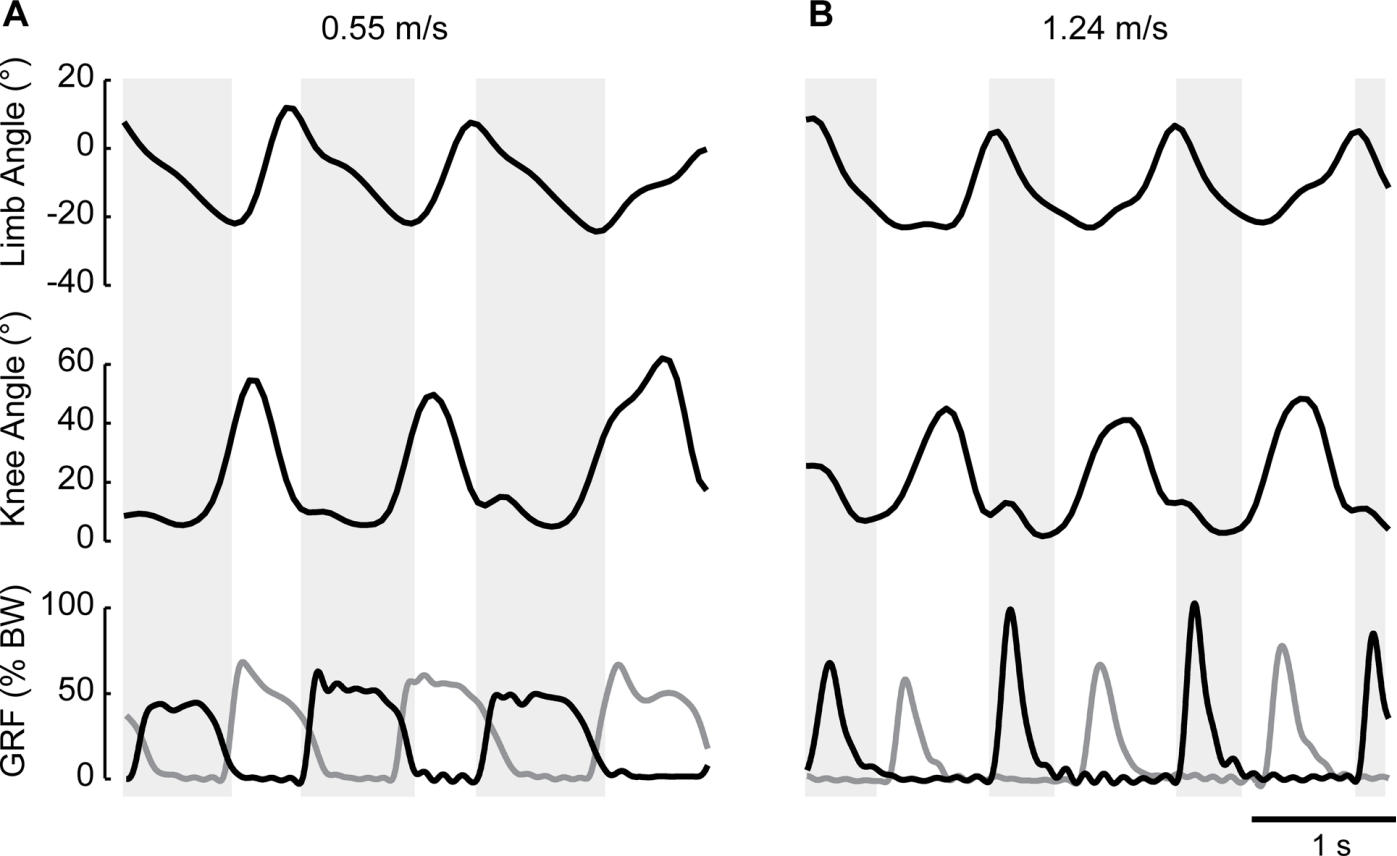
Kinematic and force data from an infant stepping at slow (A) and fast (B) speeds. Black lines show data from right leg; grey lines show force traces from left leg. Grey boxes show the duration of right leg stance phase and white spaces in between boxes show right leg swing phase. Limb angle is defined in Fig 2. Positive limb and knee angles indicate flexion. GRF: Ground Reaction Forces; BW: Body Weight.

In this experiment, our objective was to test whether infants transition from walking to running in response to changes in afferent input (i.e. a faster moving treadmill belt). Given that infants were supported during stepping, the presence of a flight phase is not necessarily indicative of running, as it is in adults. Therefore, we examined additional kinematic parameters that demarcate walking and running in adults, such as knee angle, support length, limb angle, and center of mass displacement. Fig 3 shows kinematic data and forces during sequences of stepping at slow (0.55m/s) and fast (1.24m/s) speeds in an example infant (aged 10 months). The stepping pattern at the slow speed showed periods of double support, as can be observed in the force traces (overlapping forces between the right and left sides); stepping at the fast speed shows periods of flight. Despite the appearance of flight in the fast trial, kinematic measures (knee and limb angle) are similar across speeds.

**Fig 4:**
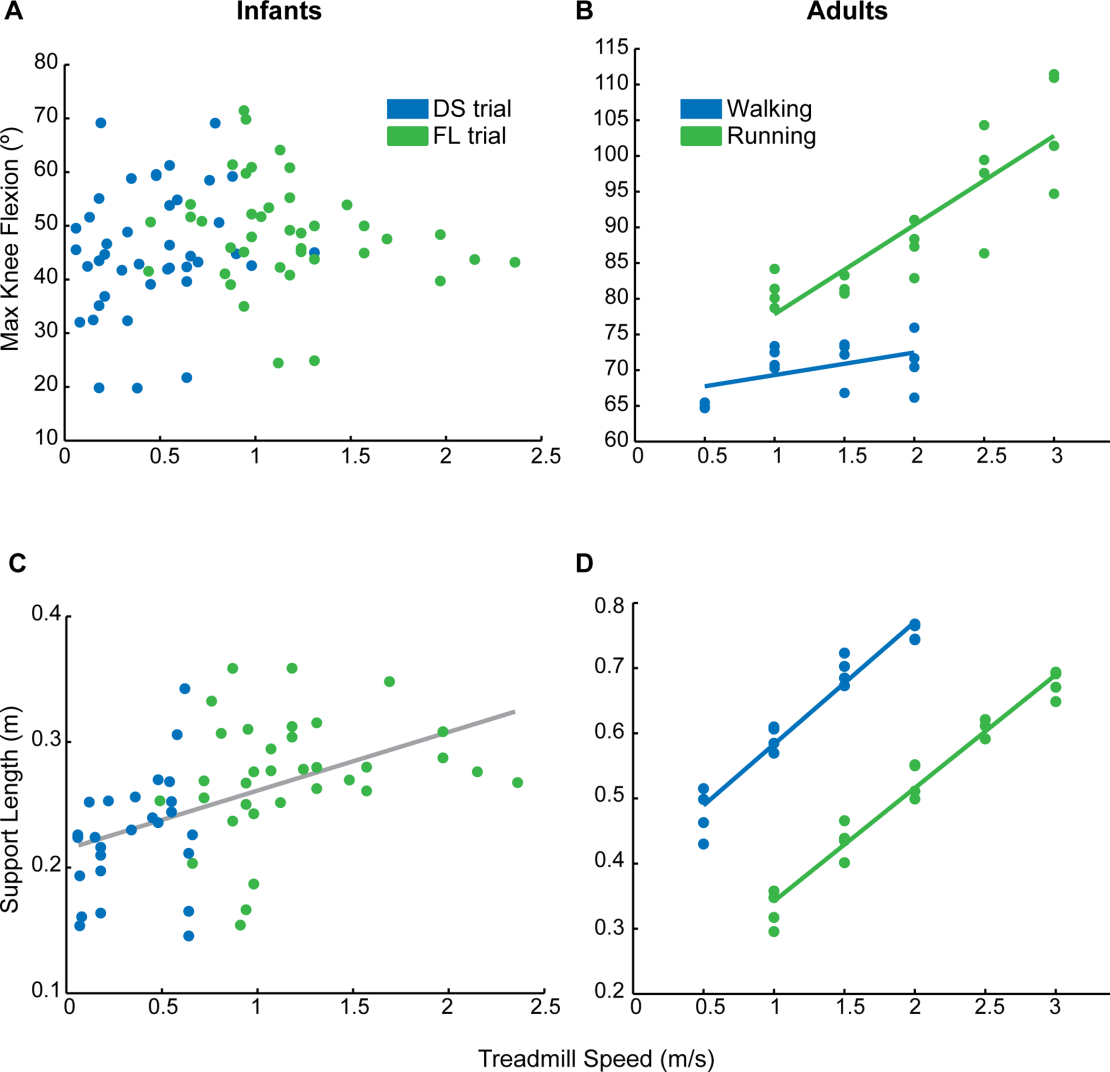
Adaptation to treadmill speed in infants and adults: changes in maximum knee flexion angle (A, B) and support length (C, D). In infants (left column), trials in which the majority of strides had a period of double-support (DS) are in blue; trials that had mostly flight (FL) strides are in green. For adults (right column), walking and running trials are shown in blue and green, respectively. Lines were fit to each dataset, except the infant knee angle data (A), which were not significantly correlated with treadmill speed (r^2^ <0.03). For infant support length (C), a line was fit to all data (walking and running trials) since the correlation between treadmill speed and support length was only significant when all data were included.

In adults, the amplitude of knee flexion during swing changed differently with speed depending on whether the individual was walking or running. During running, knee flexion increased with speed (r = 0.93), and values exceeded 90° of flexion at faster speeds. During walking, knee flexion angle changed less across different speeds, maintaining a relatively constant value of 65–75° of flexion (r = 0.52) (Fig 4B, also see [36]). Fig 4A shows maximum knee flexion during the swing phase across different treadmill speeds in infants. Average values during trials in which the majority of strides had a period of double-support are shown in blue; flight trials are shown in green. There was no significant correlation between treadmill speed and knee angle for infants, for either double-support or flight trials (r < 0.1). Therefore, unlike adult running, infants did not increase knee flexion with treadmill speed in trials with flight strides.

Fig 4C and 4D show support length (distance traveled by the foot during stance phase) versus treadmill speed in infants and adults, respectively. During adult locomotion, support length generally increased with treadmill speed (Fig 4D, also see [36]), similarly to what we observed in infants (Fig 4C) (r > 0.98 for adult walking and running; r = 0.50 for infants, collapsing across walking and running trials). However, a longer support length was used during walking, compared to running at an equivalent speed (Fig 4D, also see [36]). Infants, on the other hand, showed little difference in support length between double-support and flight trials at similar speeds (Fig 4C).

**Fig 5:**
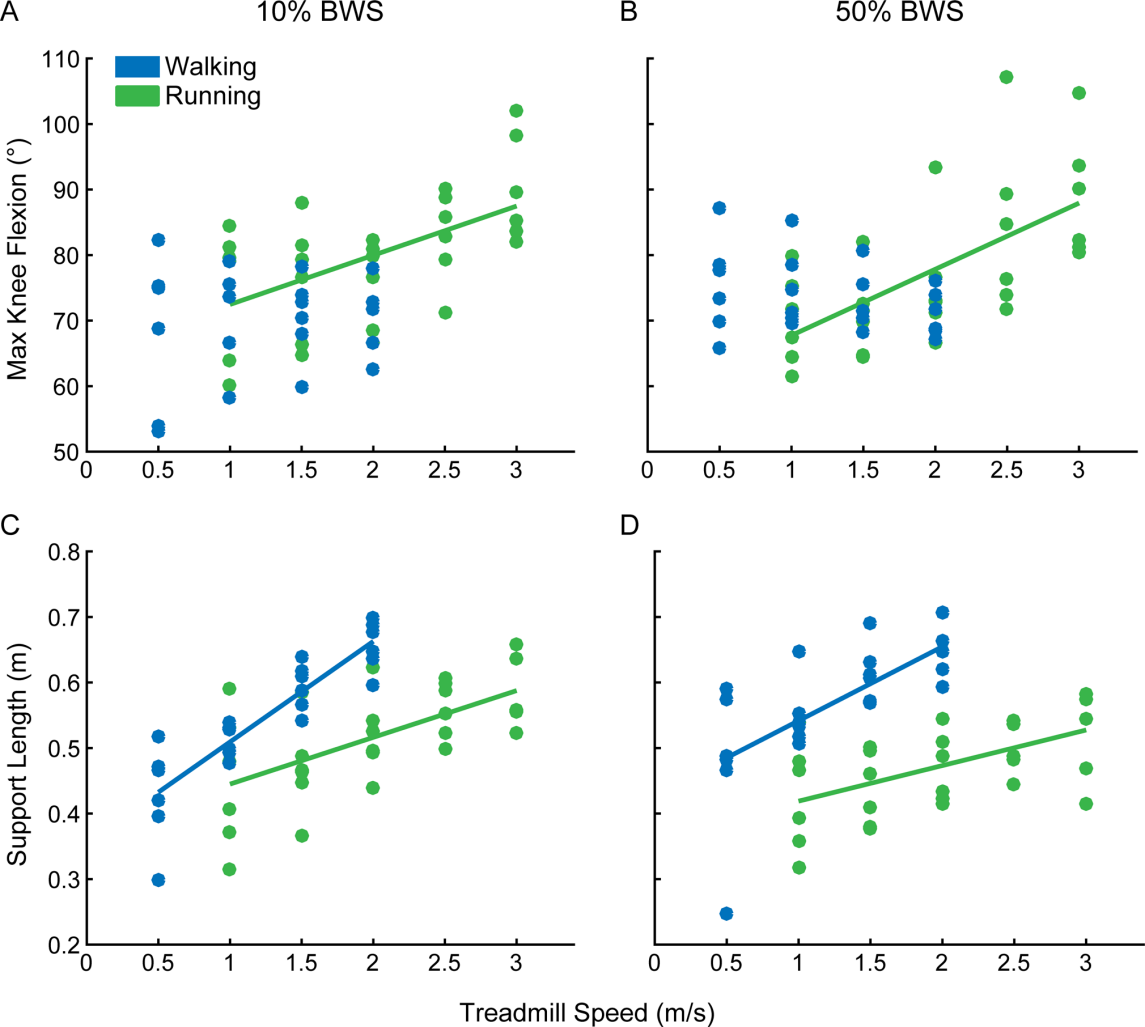
Adaptation to treadmill speed in adults who bore 90% body weight (i.e., 10% body weight support; A, C) and 50% body weight (B, D). Data are as shown in Fig 4. Lines were fit to each dataset when changes in the parameter (max knee flexion: A, B; support length: C, D) were significantly correlated with gait speed. Pearson correlation coefficients are reported for walking and running data in blue and green text, respectively (asterisks: p<0.05). BWS: Body Weight Support.

One difference in the experimental protocol used for infants and adults in this study is that the infants were provided with body weight support, whereas the adults bore full body weight. To determine whether body weight support contributed to the differences we observed between adult walking and running, we collected data from six adults who walked and ran at speeds ranging from 0.5–3.0m/s while bearing 90% body weight (i.e. 10% Body Weight Support; BWS) and while bearing 50% body weight. Fig 5A and 5B show significant correlations between knee flexion angle during swing phase and running speed during both body weight support conditions (r > 0.5). Similar to the no body weight support condition (Fig 4B), maximum knee flexion angle was relatively constant across speeds during the walking trials (r ≤ 0.1). Therefore, the observation that there is a stronger correlation between knee flexion angle and gait speed during running trials versus walking trials is true regardless of the body weight support condition. We also found similar trends in the correlation between support length and gait speed across all conditions of body weight support. Fig 4D (0% body weight support), Fig 5C (10% body weight support) and 5D (50% body weight support) all show significant correlations between treadmill speed and support length during both walking and running trials (p ≤ 0.001). Additionally, support length was consistently reduced during running trials compared to walking trials at the same speed in all body weight support conditions (Figs 4D, 5C and 5D; also see [36]).

**Fig 6:**
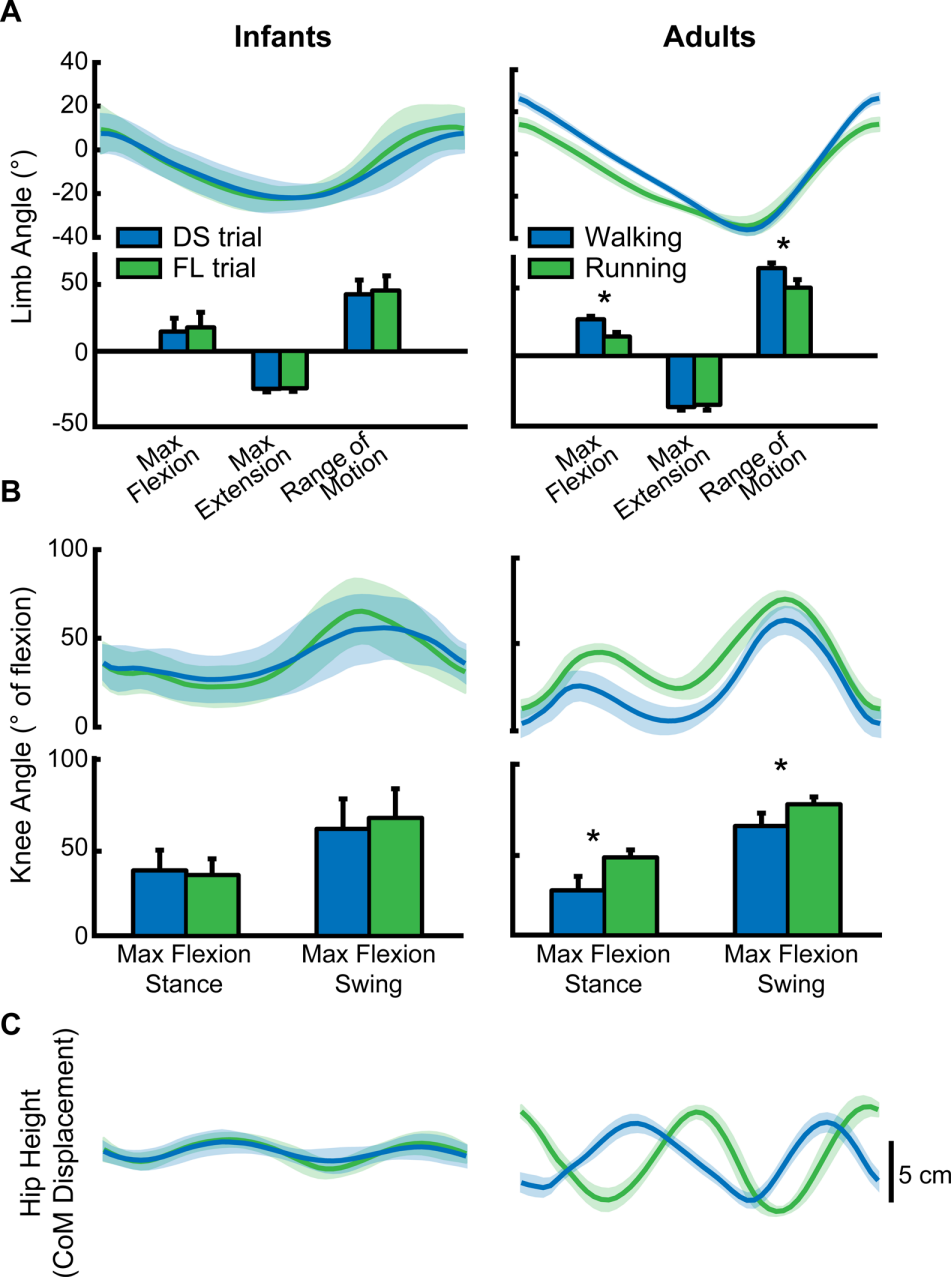
Comparison between kinematic variables known to change at the adult walk-run transition. Each infant plot (left) shows data from a subset of 10 infants who had at least one transition trial, defined as a trial in which 30–70% of strides had a period of flight. Double-support (DS-blue) and flight (FL-green) strides were averaged separately. Adult plots (right) show data from 6 adults who walked (blue) and ran (green) at the same speed (2.0m/s). **(A)** Top: averaged limb angle traces (± SEM across infants) for DS/walking and FL/running strides (foot-contact to foot-contact; normalized to the same cycle duration). Bottom: averaged maximum limb flexion, extension, and range of motion (* p<0.05). **(B)** Knee angle is expressed as degrees of flexion (0° = straight leg). Data are as shown in (A). **(C)** Hip height was used as an estimate of Center of Mass (CoM).

In this study, we also wished to directly compare double-support and flight strides taken at similar treadmill speeds in infants, to provide an analogous comparison to adult walking and running at similar speeds. To this end, we identified a subset of ten infants who performed at least one successful “transition trial”, defined as a trial in which 30–70% of strides showed a period of flight. For each of these trials, kinematic data from strides that showed a period of double-support were averaged separately from flight stride data as shown in Fig 6 (left column). Data from seven adults who performed walking and running at the same speed (2.0m/s) were collected and used for comparison (Fig 6, right column).

Fig 6A (top) shows limb angle averaged across double-support (blue) and flight (green) strides for infants (left). Adult limb angle data averaged across walking (blue) and running (green) are shown on the right. Shaded regions indicate one standard error. Each limb angle trace starts and ends with foot contact. In adults, the transition from walking to running was marked by a decrease in limb angle flexion at heel strike, and an overall decrease in limb range of motion (Fig 6A, bottom right). In contrast, limb angle excursion was very similar during double-support and flight strides in infants (Fig 6A, left). Similarly, clear differences were observed in adult knee angle between walking and running; when running, adults showed greater knee flexion during stance and swing phase (Fig 6B, right). Infant knee angle flexion, however, was unchanged between double-support and flight strides (Fig 6B, left).

The final parameter we examined was hip height, which was used to estimate displacement of the center of mass. Differences in adult walking and running were obvious. In walking, hip height peaked at midstance on the right side (first peak) and then at midstance on the left side (second peak), which suggests an “inverted pendulum” mode of progression (Fig 6C, right). During running, peaks occurred at the end of stance on each side, when the body is launched in the air, suggesting a spring-like gait [38, 46, 47]. Shifts in the timing of center of mass displacement were quantified as the phase lag at the peak cross-correlation between double-support/walking and flight/running traces. For adults, the mean value was 0.37 ± 0.03 standard deviations (range: 0.32–0.40), indicating that the signals were offset by 37% of a cycle. Infant hip height data showed very little offset in timing between double-support and flight strides (Fig 6C, left–phase lag = 0.09 ± 0.18). The phase lag between walking and running center of mass displacement was significantly greater in adults than infants (ρ < 0.001).

**Fig 7:**
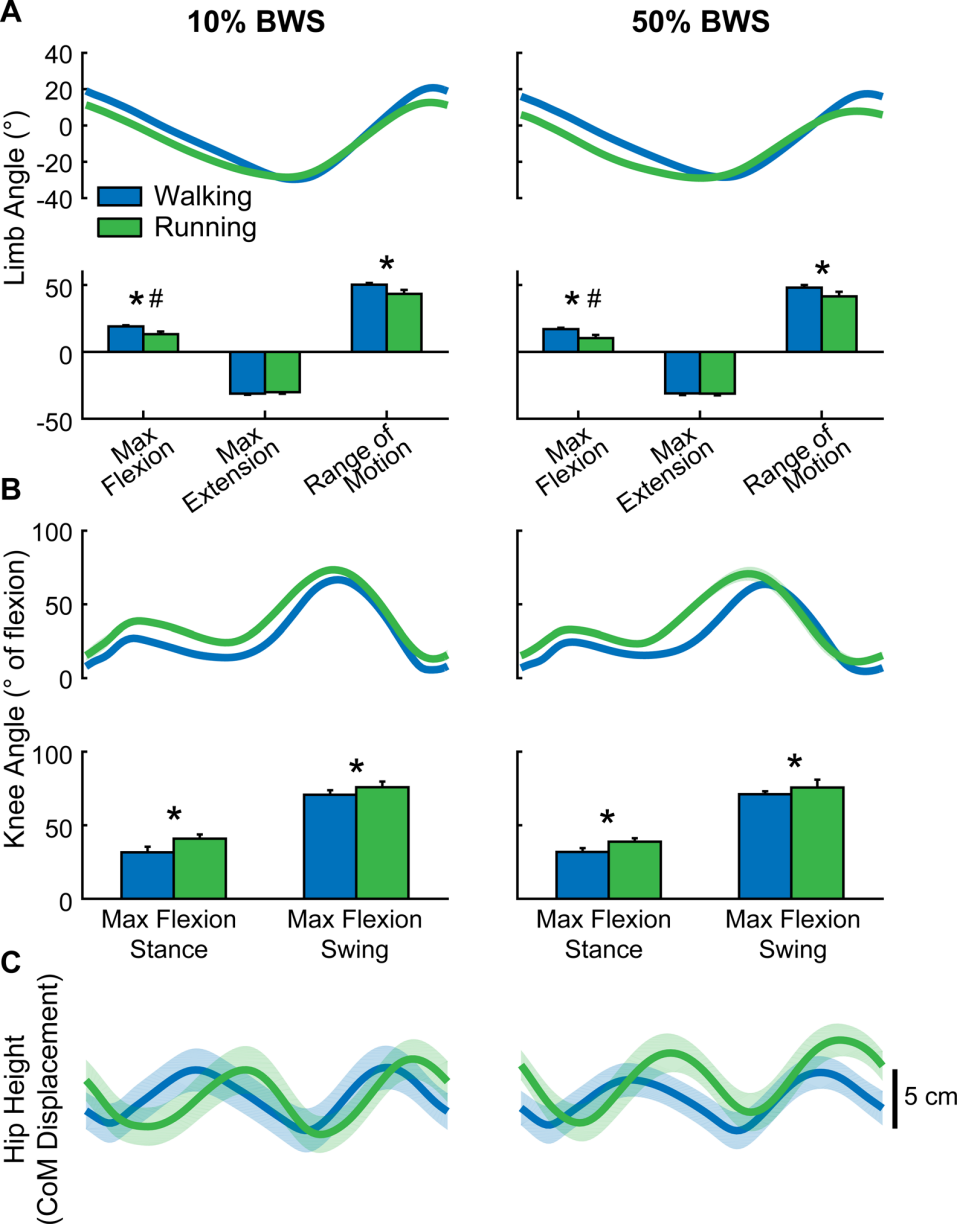
Changes in kinematic variables at the walk-to-run transition in adults who bore 90% body weight (i.e., 10% body weight support; left column) and 50% body weight (right column). Data are as shown in Fig 6. Asterisks (*) show significant main effects for gait (walk versus run) for each measure; hash marks (#) show significant main effects for body weight support (10% versus 50%). BWS: Body Weight Support.

We also examined the changes in the parameters shown in Fig 6 under different body weight support conditions in adults (Fig 7). Adults walked at 2.0 m/s while bearing 90% (10% BWS) or 50% (50% BWS) of their body weight. Although adults were provided with body weight support during these trials, we still observed significant changes in maximum limb flexion angle and limb range of motion between walking and running trials (Fig 7A; asterisks mark main effects for gait type, ρ < 0.01). Furthermore, we found that adults with body weight support still showed differences in knee flexion angle between walking and running trials (Fig 7B, ρ < 0.05). Both of these findings parallel the differences we reported between walking and running in adults without body weight support (Fig 6A and 6B, right column). Maximum limb flexion was reduced during 50% BWS trials compared to 10% BWS trials (main effect for BWS shown by # in Fig 7A, ρ = 0.02). None of the other limb or knee angle measurements were affected by the amount of body weight support (ρ > 0.2).

The phase lag between walking and running vertical center of mass displacement was not significantly different between trials with 10% and 50% BWS (ρ = 0.40). However, we also noticed that the phase lag values during 10% BWS trials (0.25 ± 0.19, mean ± s.d.) and during 50% BWS trials (0.19 ± 0.21) appeared to be smaller and more variable than the phase lag values obtained from adults with no body weight support (0.37 ± 0.03). We examined individual subject phase lag values for the adults tested with BWS (Table 1) and discovered that phase lags varied between 0 (i.e., no significant shift in center of mass displacement between walking and running trials) and 0.44 (i.e., a 44% offset in center of mass displacement between walking and running trials). In contrast, phase lag in center of mass displacement for adults without body weight support was more consistent (range: 0.32–0.40). Table 1 also displays phase lag values for infants included in Fig 6C (right column) and shows that, similar to the adults with BWS, the infants show phase lags between 0 and 0.44. Interestingly, phase lag values for infants and adults with body weight support are either grouped around 0 or 0.4, indicating a bimodal distribution. Most of the infants (8/10) show phase lag values near 0, compared to 2/6 adults in the 10% BWS condition and 3/6 adults in the 50% BWS condition.

**Table 1:** Phase differences in vertical center of mass displacement during walking (double support strides) and running (flight strides) in infants and adults with body weight support.

Participant	Age (mo)	Speed (m/s)	BWS (%)	Phase lag
I1	8.9	1.1	6.3	0
I2	10.3	1.2	43.1	0
I3	9.7	0.7	68.6	0
I4	10.4	0.9	57.1	**0.44**
I5	9.1	0.9	63.5	**0.4**
I6	9.1	0.9	57.6	0
I7	10.3	1.0	44.7	0
I8	9.1	0.5	64.8	0
I9	10.4	0.9	45.4	0
I10	9.5	0.8	—-	0.02
A1	Adult	2.0	10 / 50	**0.38** / **0.44**
A2	Adult	2.0	10 / 50	**0.38** / 0
A3	Adult	2.0	10 / 50	0 / 0
A4	Adult	2.0	10 / 50	**0.35** / **0.35**
A5	Adult	2.0	10 / 50	**0.37** / **0.32**
A6	Adult	2.0	10 / 50	0 / 0

BWS, body weight support; mo, months. Adult phase lags are shown for trials with 10% and 50% BWS, separated by a forward slash (/). Bolded values highlight phase lags between 0.32–0.44, which indicate differences in vertical center of mass displacement timing between walking (double support) and running (flight). We did not obtain body weight support measurements from one infant (I10).

## Discussion

A critical survival skill for most terrestrial, legged animals is the ability to locomote at different speeds. Fast gaits can be necessary for securing food, avoiding becoming food, and evading other life-threatening situations; slower gaits are often used during migrations and grazing or gathering behaviors. A common strategy to minimize energy expenditure across a range of speeds is to transition between different forms of gait [48]. Such gait transitions are marked by a discontinuous change in locomotor coordination. Quadrupedal animals like horses and cats tend to make gait transitions that involve changes in interlimb coordination to walk, trot, and gallop [28, 49]; large, bipedal animals like ostriches and humans tend to alter intralimb coordination to walk or run [40, 50–52].

While infants are capable of altering interlimb coordination in order to step, jump, or even cycle the two legs at different rates (i.e. on a split-belt treadmill) [24, 30–35], we did not find evidence that infants altered intralimb coordination to produce distinct walking and running patterns. Infants were clearly capable of accommodating a remarkably wide range of speeds, but the gait that was used at fast speeds was a simply a faster version of the gait used at slow speeds.

### How fast were infants capable of stepping?

The infants in this study could accommodate a remarkably wide range of speeds, from 0.06 to as high as 2.36m/s. For reference, preferred walking speed in young adults is around 1.1–1.2m/s [40, 41] and the walk-run transition occurs around 1.88–2.35m/s [42–45]. This means that the four successful infant trials that we collected above 1.75m/s (Fig 2A) occurred near or above speeds at which adults would normally transition to running.

Given the smaller stature of infants compared to adults, it may be reasonable to assume that treadmill speeds in the current experiment surpassed that at which a gait transition should have occurred in infants. A more accurate way to estimate transition speed relative to size is to use the Froude number, a dimensionless parameter used to normalize the same pendulum-like motion (i.e., walking) in subjects of different height and under different loading conditions [40, 45, 48, 51, 53, 54]. The Froude number is calculated by the equation $Fr=\frac{v^{2}}{gL}$ where *v* is the speed of locomotion, *g* is the acceleration of gravity, and *L* is leg length. In humans, the walk-run transition occurs around *Fr* = 0.5 [40, 45, 48, 53]. It is therefore possible to estimate the velocity at which a gait transition should have occurred in infants by using the equation $v=\sqrt{Fr\cdot g\cdot L}$ where *Fr* = 0.5. Average leg length for 9–10 month old infants (the mean age of our infant participants) is 25cm [55]. Infants supported, on average, 47% of their body weight, thus *g* = 0.47 × 9.81*m* / *s*^2^. This gives a value of *v* = 0.76*m* / *s* —well within the range of speeds tested here. Altogether, this suggests that we did not observe gait transitions because infants were not capable of making such transitions under these conditions, and not because we did not push the treadmill speed high enough. Note that a period of flight started appearing around the speed at which *Fr* = 0.5, yet other expected intralimb coordination changes accompanying a gait transition were not observed.

### Changes in gait to accommodate speed in infants

Without making gait transitions, how did infants accommodate the range of treadmill speeds? We discovered that they adjusted gait parameters similarly to cats and human adults when walking at different speeds. The relationship between stride cycle duration and treadmill speed is fit by a power function, as previously described by Yang and colleagues [12], the parameters of which are very similar to those reported for cats [28]. Changes in stride cycle duration were largely driven by changes in the stance or support phase duration, which is also consistent with findings from cats [28, 56], adult humans [36], and spinal cord injured humans [10]. In this study, we also discovered that when stride cycle duration is very short (cadence is high), the correlation between swing and stride cycle duration increases. This supports a CPG model where either the flexor or extensor phase can dominate (as proposed in [57]). Overall, the similarities between how human infants, human adults, and cats with and without spinal lesions adjust gait cycle and phase durations in response to changes in sensory input suggest that these modifications can be coordinated by spinal pattern generators interacting with peripheral feedback.

Also similar to adults is our finding that human infants incorporated a period of flight into stepping at speeds exceeding ~1.0m/s. While flight is commonly used to define a running gait in adults, here we argue that this definition is insufficient for identifying gait transitions in infants. For adults supporting all of their weight, achieving a period of flight requires the ability to launch the body into the air at the end of each stance phase. Infants, on the other hand, were supported by an experimenter during stepping. They were encouraged to bear as much weight as possible (47% body weight, on average); nonetheless, it may be mechanically easier to achieve a period of flight, since the experimenter would not let them fall. We therefore also examined measures of intralimb coordination that abruptly change at the adult walk-to-run transition, specifically knee flexion angle during swing and a decrease in support length.

Changes in intralimb coordination at the walk-to-run transition are related to a shift in the mechanics of progression. Walking is well-described by an inverted pendulum model in which changes in potential and kinetic energy of the center of mass are in opposite phases [38, 47, 58]. In the stance phase, the center of mass moves over a rigid strut (the leg). Once the center of mass has reached its maximum height at mid-stance (i.e., maximum potential energy), the body falls forward with gravity (i.e. losing potential energy phase while gaining kinetic energy) and is caught by heel strike on the opposite side; the displacement of hip height, as an estimate of center of mass displacement, is shown in Fig 6C (adult walking). Heel strike in walking occurs when the knee is near full extension, resulting in a large support length (Fig 4D). Knee flexion during swing is minimized to conserve energy (Fig 6B). In contrast, running is described by a bouncing gait model in which potential and kinetic energy of the body rise and fall at the same time [38, 47, 59]. In other words, the bouncing gait allows storage of spring potential energy during stance (i.e., in muscles and tendons), which is released and used to achieve the height during the swing phase (Fig 6C, adult running). Foot contact occurs with the knee more flexed than during walking to reduce ground reaction forces and to allow the storage of spring potential energy in the leg muscles; this is associated with a decrease in support length (Fig 4D). Since swing needs to be fast, there is greater flexion of the knee to reduce the moment arm (Fig 6B).

Across the wide range of speeds tested, infants did not change intralimb coordination in a manner that would suggest a gait transition. There were no distinct shifts in knee angle or support length. As a group, infants also did not show changes in vertical center of mass displacement that would indicate a change in mechanics of progression. From the hip height traces, it appears that a peak in center of mass displacement occurs near mid-stance, which would suggest that infant stepping is best described by an inverted-pendulum model (i.e. walking). However, other aspects of infant stepping more resemble adult running–the knee is flexed at heel strike and throughout stance (Fig 6B), and limb flexion angle is reduced at heel strike (Fig 6A), similar to adult running. Furthermore, other work has shown that infants aged 4–12 months tend to lack a true heel strike and often walk on their toes [60], which is also more similar to adult running. Based on these data, we suggest that infants show neither a mature walking pattern, nor a mature running pattern. Rather, they use a pattern composed of some aspects of each, and this is the pattern that they use across a range of different speeds.

### Effects of body weight support on the emergence of gait transitions

Recently, it has been shown that the provision of body weight support affects the walk-to-run transition in adults: this transition progresses more gradually when adults walk in simulated reduced gravity conditions (<0.38g), compared to walking in normal conditions [45, 61]. To determine if body weight support could influence the measures of interest in the current study, six adults were tested across a range of speeds spanning the walk-to-run transition with 10% and with 50% body weight support (Figs 5 and 7). Intralimb coordination in adults who walked and ran with body weight support (Fig 5) appeared to be more variable than the data from adults who walked and ran without body weight support (Fig 4). Regardless, body weight support did not conceal key changes in intralimb coordination that occur at the walk-to-run transition in adults. As in normal loading conditions, knee flexion angle during swing increased with treadmill speed during running, whereas knee flexion remained relatively constant during walking trials in the body weight support trials (Fig 5A and 5B). A direct comparison of walking and running at the same speed showed that knee flexion angle was greater during running trials regardless of body weight support (compare Figs 6B and 7B). Support length was shorter during running compared to walking at the same speed across all loading conditions (Figs 4D, 5C and 5D). Finally, we found that body weight support did not affect the reduction in limb flexion angle that we observed at the walk-to-run transition (Figs 6A and 7A). Therefore, up to 50% body weight support did not completely erase signs of a gait transition in adults.

On the other hand, even low amounts of body weight support could alter the timing of center of mass displacement in some, but not all, adults. All of the adults who walked and ran under normal loading conditions showed a timing shift in vertical center of mass displacement: the pattern of center of mass displacement was offset by ~40% of a cycle when comparing walking and running at the same speed (Fig 6C, Table 1). When body weight support was introduced, this shift in center of mass displacement timing was absent in 2/6 adults in the 10% BWS condition and 3/6 adults in the 50% BWS condition (Fig 7C, Table 1). This suggests that some adults did not fundamentally change the mechanics of progression when they were asked to walk or run at 2.0 m/s with body weight support. This finding agrees with other work that has suggested that loading at early stance is a critical for triggering a transition to running in adult humans [62, 63].

Did the body weight support during infant stepping mask signs of the walk-to-run transition? One possibility is that the person supporting the infant could have imposed movements on the infant, thus externally influencing vertical displacement of the center of mass. While we cannot rule this out, it is interesting that our adult and infant participants showed a bimodal distribution of center of mass displacement phasing: center of mass displacement was either offset by ~40% of a cycle between walking and running strides, or there was no shift (~0% phase lag) (Table 1). If the person holding the infant imposed his/her own movements on the infant, an even distribution of phasing values across the full range (0–1) might be expected.

Perhaps a larger concern pertains to whether the body weight support experienced by the infants masked the walk-to-run transition in the same way that body weight support in adults partially masked the walk-to-run transition. Again, we cannot rule out this possibility. However, note that body weight support in adults only concealed abrupt changes associated with the walk-to-run transition in the center of mass measure. Other differences in intralimb coordination remained. As a group, infants neither changed center of mass nor intralimb coordination in a way that resembled a mature walk-to-run transition.

How significant are the findings from the two infants who showed timing shifts in center of mass displacement (Table 1)? Little can be inferred from the data of two infants since infant locomotion is variable. Additional data are needed to determine whether this finding is valid and reliable, and also to compare infants who showed timing shifts to those who did not. The weight of the evidence right now suggests that, as a group, the infants did not show evidence of distinct walking and running gait patterns.

### Development of gait and gait transitions in humans and other animals

As in human development, cats and rats are not born with the ability to walk independently. The development of locomotion matures slowly over the first postnatal weeks. Rat pups, for example, do not walk independently before post-natal day 14 (PN-14) [64, 65]; kittens walk “imperfectly” at PN-31 and normal walking appears at PN-44, on average [66]. Before the onset of independent locomotion, these neonatal animals can coordinate locomotor movements when supported by the tail on a moving treadmill [56] or when stimulated by excitatory neurotransmitters [67] or an olfactory stimulus [68]. Neonatal animals display both alternating (i.e., walking) and synchronous (i.e., galloping) interlimb coordination even during air-stepping, when the limbs are completely unloaded [67]. The alternating pattern may appear slightly earlier than the synchronous pattern in neonatal rats (PN-5 for alternating; PN-10 for synchronous); nonetheless, both patterns emerge before these animals are independent walkers [67]. Following complete thoracic spinal cord transection at PN-6 –PN-17, kittens are still capable of producing different forms of interlimb coordination, and can walk, trot and gallop [5]. Neonatal rats can also produce stepping movements with good interlimb coordination following spinal cord transection, although aspects of intralimb coordination–specifically, coordination of knee and ankle actions–may not completely recover [69]. Therefore, evidence from young quadrupedal animals suggests that different forms of interlimb coordination can be coordinated by spinal pattern generators and that experience with independent locomotion is not essential for the emergence of these behaviors. Interestingly, refinement of intralimb coordination may require descending input from higher centers.

Similar to young quadrupeds, human infants do not need practice with independent stepping in order to produce different forms of interlimb coordination with the lower limbs. However, the maturation of locomotor patterns, like walking and running, which require modifications in intralimb coordination, may proceed at a slower rate. Whitall and Getchell [46] found that newly-independent walkers do not use different energetic strategies for strides with double-support and strides with flight; in fact, only after 9.5 months of independent walking were separate walking and running strategies observed. The emergence of distinct walking and running gaits coincided with the development of stable coordination around the ankle, which suggests that the ability to produce and/or regulate force by ankle extension may be the rate-limiting step for the emergence of running [46]. This aligns with other reports showing that coordination about the ankle is immature in early life; infants tend to walk on their toes [14, 60] and a true heel-strike is not observed until 2 years of age [70]. This immature coordination about the ankle, specifically the immaturity of ankle extensor control, may prohibit the emergence of a running gait.

Interestingly, walking and running in non-human bipeds do not mature at the same rate. One to two days post-hatching, chicks show a running pattern similar to the pattern observed at 14 days post-hatching; in other words, the running pattern was relatively mature very early in life. The walking pattern, however, was immature at post-hatching day 2 compared to day 14 [71]. Specifically, there were significant differences in how the ankle and knee were coordinated at day 2 and 14 [71]. Overall, our data along with findings in other experimental models suggest that young bipedal animals do not show mature walking and running patterns.

## Conclusions

It has been proposed that loading conditions trigger the transition to running in human adults [62, 63]. If so, why would we expect gait transitions in weight-supported infants? First, although there is almost certainly a biomechanical component to the adult walk-to-run transition, there are probably differences in the neural control of walking and running as well. Even when we diminished the biomechanical drive to transition to a running gait by supporting the adults’ body weight, we did not completely erase signs of a gait transition. The remaining differences between walking and running in body weight support conditions may reflect the retrieval of separate motor patterns for walking and running. Ogawa et al [72, 73] also found evidence supporting distinct neural control for the two gait forms: using a well-studied split-belt treadmill paradigm, they found that when human adults were trained on the split-belt treadmill while walking, the learned motor behavior did not transfer completely to running and vice-versa, suggesting limited overlap in the neural control of the two forms of gait. In the present study, we found little evidence for distinct walking and running patterns in human infants, even though these infants sometimes gave the superficial appearance of running at high speeds (S1 Video). Taken together, the evidence suggests that walking and running are controlled by distinct neuronal circuits that are not mature in infancy. The emergence of mature walking and running patterns likely requires neuromuscular maturation, particularly maturation of the control of ankle extensor muscles, and a period of learning during independent, weight-bearing locomotion.

## Supporting Information

S1 Fig Histograms showing interlimb coordination of analyzed strides at different speeds. Phasing was calculated as the time of right side foot contact relative to the left side stride cycle, and expressed as a percentage. Values near 50% indicate alternating stepping and values near 0 or 100% indicate synchronous coordination, like bouncing. A-D show histograms for progressively faster speed ranges.
(TIF)

S1 Video Example infant (female, 10 months old) stepping at 2.0 m/s. (MPG)
