# Supplementary figures and images for: Gait Transitions in Human Infants: Coping with Extremes of Treadmill Speed
Source: PLoS One. 2016 Feb 1;11(2):e0148124. doi: 10.1371/journal.pone.0148124 (PMC4734668; doi:10.1371/journal.pone.0148124)

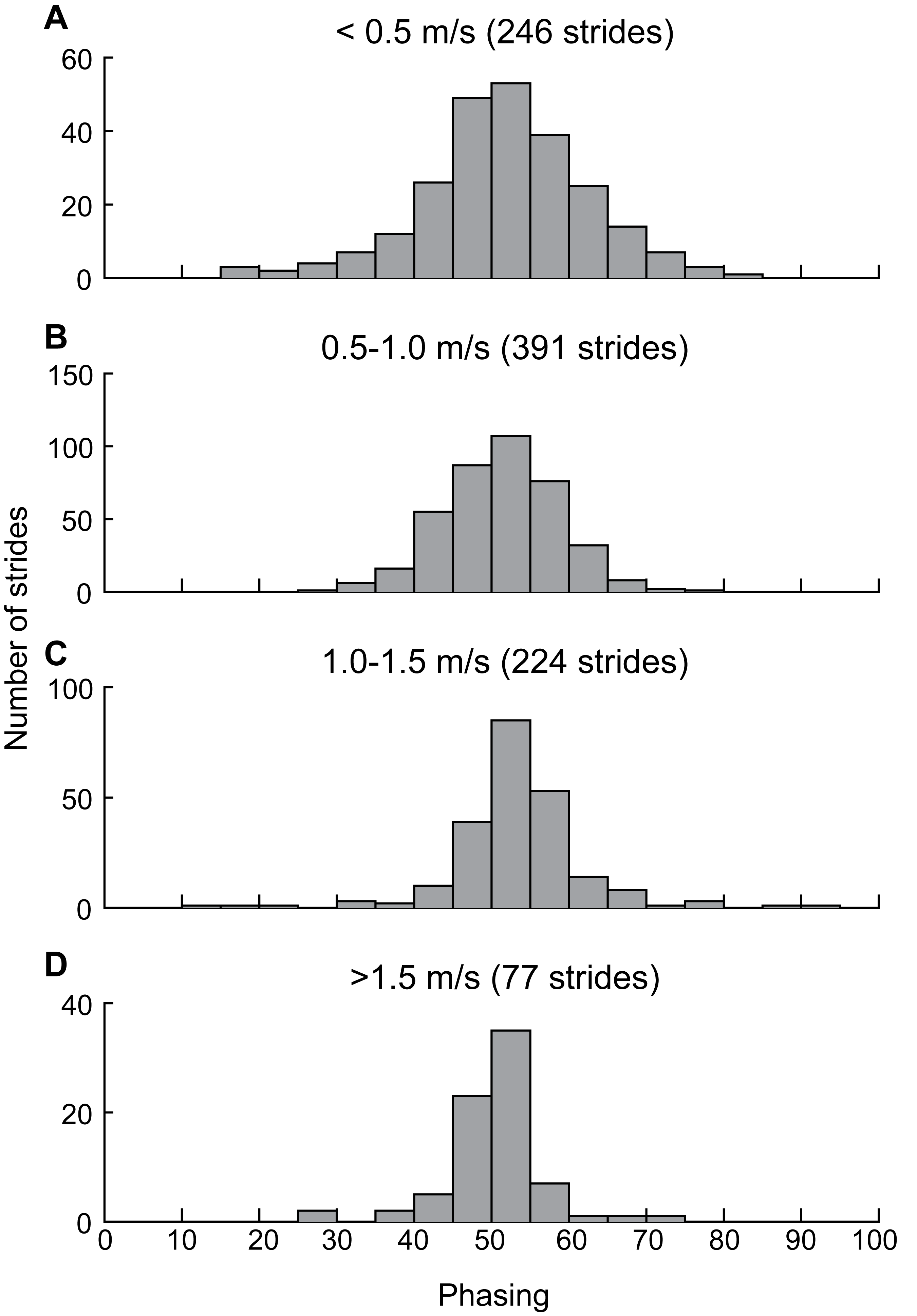

Supplement: S1 Fig — Phasing was calculated as the time of right side foot contact relative to the left side stride cycle, and expressed as a percentage. Values near 50% indicate alternating stepping and values near 0 or 100% indicate synchronous coordination, like bouncing. A-D show histograms for progressively faster speed ranges. (TIF) [file pone.0148124.s001.tif]
